# Identification of novel antimicrobial peptide from Asian sea bass (*Lates calcarifer*) by *in silico* and activity characterization

**DOI:** 10.1371/journal.pone.0206578

**Published:** 2018-10-26

**Authors:** Behrouz Taheri, Mohsen Mohammadi, Iraj Nabipour, Niloofar Momenzadeh, Mona Roozbehani

**Affiliations:** 1 Department of Medical Laboratory Sciences, School of Paramedicine, Ahvaz Jundishapur University of Medical Sciences, Ahvaz, Iran; 2 The Persian Gulf Marine Biotechnology Research Center, the Persian Gulf Biomedical Sciences Research Institute, Bushehr University of Medical Sciences, Bushehr, Iran; 3 PhD Students of Medical Parasitology School of Medicine, Iran University of Medical Sciences, Tehran, Iran; Bose Institute, INDIA

## Abstract

**Background:**

The global crisis of antibiotic resistance increases the demand for the new promising alternative drugs such as antimicrobial peptides (AMPs). Accordingly, we have described a new, previously unrecognized effective AMP, named dicentracin-like, from *Asian sea bass* and characterized its antimicrobial activity by comparison with moronecidin.

**Methodology/ Results:**

Gene expression analysis demonstrated the expression of dicentracin-like peptide in tissues of the immune system such as the skin and the head kidney, which is an important endocrine and lymphoid organ. Moronecidin and dicentracin-like exhibited a higher antibacterial activity against gram-positive bacteria relative to gram-negative ones, while both peptides showed a greater binding ability to gram-negative bacteria compared to gram-positive ones. This contradiction between antibacterial activity and binding affinity may be related to the outer membrane from gram-negative bacteria. Compared with moronecidin, dicentracin-like peptide showed more potent binding ability to all gram-positive and gram-negative bacteria. In addition, dicentracin-like peptide exhibited a high antibacterial activity against the investigated microorganisms, except against *Staphylococcus aureus*. A direct relationship was found between the binding affinity/cationicity and the antibiofilm activity of the peptides wherein, an elevation in pH corresponded to a decrease in their antibiofilm property. Time-kill kinetics analysis against clinical Acinetobacter baumannii isolate indicated that bactericidal effect of dicentracin-like and moronecidin at inhibitory concentration (1XMIC) was observed after 4 and 6 hours, respectively, while bactericidal effect of both AMPs at concentration of 2XMIC was observed after 2 hours. Dicentracin-like peptide showed higher inhibitory activity at subinhibitory concentration (1/2XMIC), relative to moronecidin. Compared with moronecidin, dicentracin-like peptide possessed greater binding affinity to bacteria at high salt concentration, as well as at alkaline pH; In addition, dicentracin-like exhibited a higher antibiofilm activity in comparison to moronecidin even at alkaline pH. Hemolytic analysis against human RBC revealed that hemolytic activity of moronecidin was more potent than that of dicentracin-like, which is consistent with its greater non-polar face hydrophobicity.

**Conclusions:**

In the present study, In Silico comparative sequence analysis and antimicrobial characterization led to identify a new, previously unrecognized antimicrobial function for named dicentracin-like peptide by comparison with moronecidin, representing a possible template for designing new effective AMPs and improving known ones.

## Introduction

Antibiotic agents are effective compounds to eradicate pathogenic bacteria, consequently leading to the treatment of these infections caused by these pathogens. In the past years, the emergence of multi-drug resistant pathogens has reduced therapeutic efficiency of classical antibiotics [1–3]. To fight against drug resistant pathogens, there is an urgent demand for the discovery of novel antibiotics [4]. Antimicrobial peptides have attracted considerable attention owing to their broad-spectrum antibacterial activity and their ubiquitous presence as a part of the host defense systems in both invertebrates and vertebrates [5–7]. In contrast to conventional antibiotics, most of the AMPs exert their antibacterial activity without the need for binding to a specific ligand. These AMPs disrupt membrane integrity through pore formation in the cell membrane. Furthermore, some of them can prevent proteins and nucleic acids synthesis in bacteria through inhibition of certain enzymes involved in these processes, [8, 9]. Innate immunity is very vital for vertebrates with less efficient adaptive immunities, especially those with exposure to a wide range of pathogens such as fish and amphibians. Fish as a rich sources of AMPs, expresses major classes of AMPs including pleurocidins, and piscidin [10–12]. In addition, it has been demonstrated that Fish-originated AMPs retain antibacterial properties even in high salt concentration [13]. The purification and identification of AMPs from natural sources are laborious, time consuming, and sometimes impossible due to their very low expression; accordingly, a sufficient number of animals and their tissues are required for purification of AMP [14]. To overcome these problems, the identification of AMPs-coding nucleotide sequence from small tissue-derived genomic DNA or a genomic and expressed sequence tag (EST) database is a good strategy [15]. AMPs are produced from a precursor containing a very conserved signal peptide that can be employed as a query sequence in the NCBI database to find novel AMPs [15], while the mature AMPs sequence is highly variable even in closely related species. This variability is observed in a species with different microbiota such that their sequence is under robust natural selection [15, 16]. Moronecidin, a member of the piscidin family, is an amphipathic and cationic well-known AMP that was initially isolated from the *hybrid striped bass*. Its precursor contains a signal peptide, mature piscidin-like peptide, and a C-terminal prodomain. Similar to other AMPs, the signal peptide-coding sequence of moronecidin is more significantly conserved than the two other parts [17]. In the present study, in silico comparative sequence analysis using the signal peptide of moronecidin led to identification of a new, previously unrecognized cationic peptide, named dicentracin-like, in *Asian sea bass* (*Latescalcarifer*), and afterwards antimicrobial characterization of the peptide was performed by comparison with moronecidin.

## Materials and methods

### Database searching

The signal peptide sequences of moronecidin from hybrid striped bass [18] (MKCATLFLVLSMVVLMAEPGDA, GenBankAF385583.1) was used as a query to search for the non-redundant GenBank CDS translations + PDB + SwissProt + PIR+ PRF against *Asian sea bass*(*barramundi perch*, tax id: 8187) with an algorithm parameters word size: 3, matrix: BLOSUM62, gap costs: existence 11, and extension 1 [15, 17]. The output hits were examined for the conserved signal peptide and the first case in the output was predicted as dicentracin-like(XM_018688317.1), with the highest total score (cover score: 95%; identity score:86%). The amino acid sequence of dicentracin-like (XM_018688317.1) was used as a query in BLASTP 2.2.24 + against the non-redundant Gen Bank CDS translations + PDB + SwissProt + PIR + PRF. The output revealed that the peptide was most similar to known AMPs, such as piscidin-4 and -5 precursors from the *hybrid striped bass* (GenBank ADP37959.1 and ADP37960.1). Therefore, this finding suggests that dicentracin-like (XM_018688317.1) can be a novel AMP.

### Sequence analysis

**Fig 1:**
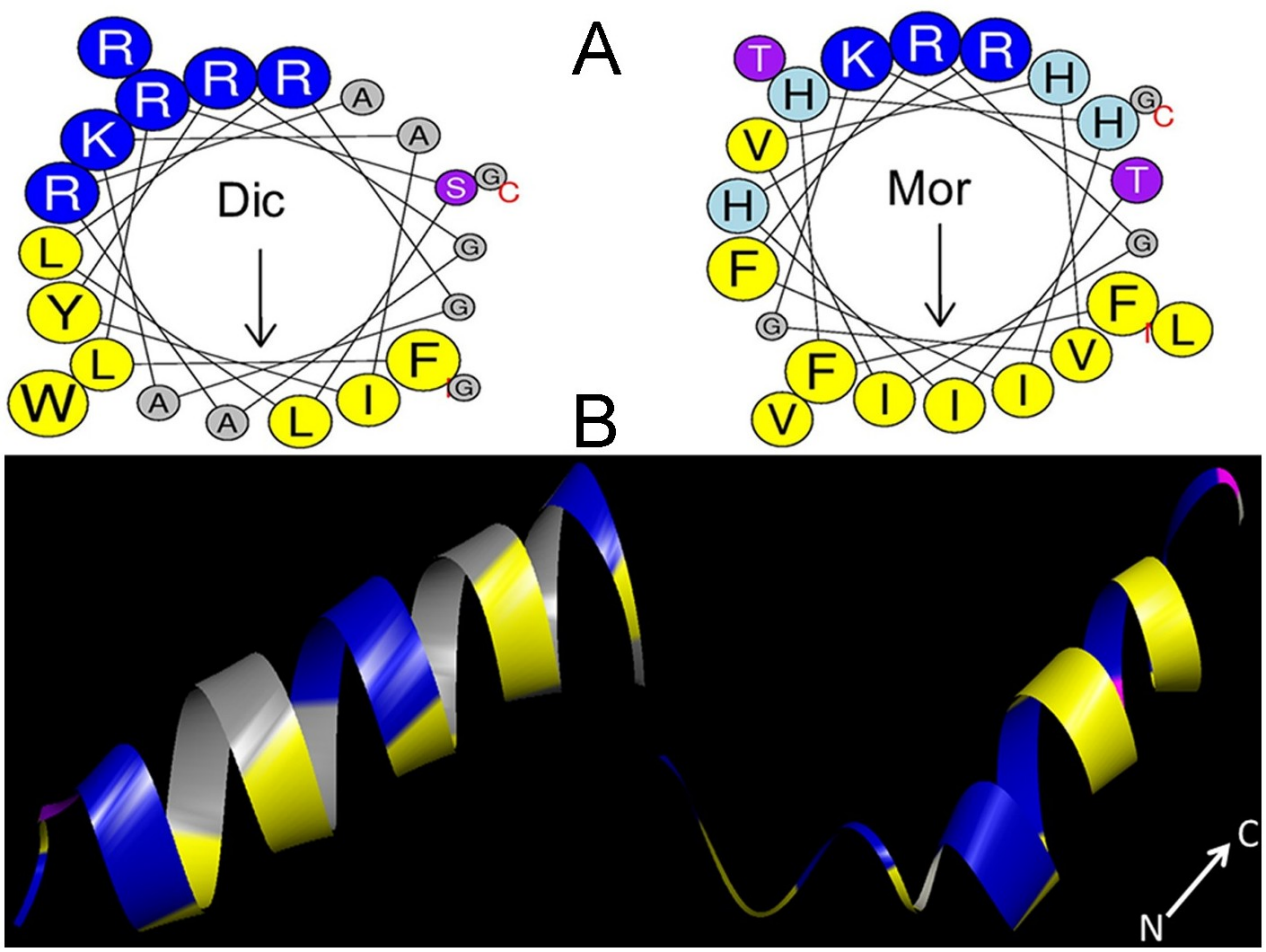
Helical wheel projections and three dimensional molecular modeling of dicentracin-like and moronecidin peptides. (A) The residue numbering starts from the N-terminus. Hydrophobic and positively charged residues are defined with yellow and blue color, respectively. (B) Three dimensional structure of dicentracin-like as predicted by I-TASSER (http://zhanglab.ccmb.med.umich.edu/I-TASSER/) and three dimensional structure of moronecidin with PDB code2 JOS as obtained from protein data bank (https://www.rcsb.org/pdb/home/home.do) and displayed with Discovery studio 3.

ORF finder (https://www.ncbi.nlm.nih.gov/orffinder/) was used to identify the protein(s)-encoded gene sequence. To identify the motifs and the signal peptide, the amino acid sequence of dicentracin-like (XM_018688317.1) was submitted in the motif finder (http://www.genome.jp/tools/motif/) and the SignalP 4.0 (http://www.cbs.dtu.dk/services/SignalP/), respectively. The position of mature AMPs on its precursor waspredicted by CAMP (http://www.camp.bicnirrh.res.in/predict/). The Antimicrobial Peptide Calculator and Predictor (http://aps.unmc.edu/AP/prediction/prediction_main.php) were used to predict antibacterial properties and identify the most similar AMPs to potential AMP. The physico-chemical properties of mature AMPs, including molecular mass (MW), isoelectric point (pI), charge, and hydrophobic score were calculated with the Protparam program from the Expasy server (http://www.expasy.ch/tools/protparam.html) (Table 1). To determine the helical wheel, the hydrophobic and hydrophilic interface on the secondary structure of peptide, its amino acid sequence was submitted in the helical wheel projection (http://rzlab.ucr.edu/scripts/wheel/wheel.cgi) [19]. The secondary structure of the peptide was predicted by an online server I-TASSER (http://zhanglab.ccmb.med.umich.edu/I-TASSER/) (Fig 1) [20].

**Table 1:** Physicochemical properties of dicentracin-like and moronecidin.

Name	Dicentracin-like	Moronecidin
Sequence	FLRSLLRGAKAIYRGARAGWRG	FFHHIFRGIVHVGKTIHRLVTG
Net charge(Ph7)/pI	6/ 12.18	3.4 / 12.01
Theoretical/Observed MW	2531.96/2531.10	2930.36/2930.50
Polar/non-polar residues (%)	50/50	54.55/45.45
Hydrophobic face residues	F G I L A A L W Y L	F L V I II F V G F
Non-polar face hydrophobicity<H>	1.25	1.49
Hydrophobicity <H>/ retention time	0.321 /20.518	0.644 /21.588
Water solubility	Good solubility	Poor solubility
Hydrophobic moment <μH>	0.55	0.55

### Ethics statement

All animal proceduresin this study were performed under protocol approved by the Animal Care and Use Committee of Bushehr University of Medical Sciences–Iran (Permit number: IR.BPUMS.REC.1395.125). The animal experiments were in accordance with the Specific National Ethical Guidelines for Biomedical Research issued by the Research and Technology Deputy of Ministry of Health and Medicinal Education (MOHME) of Iran (issued in 2005).

### Cloning and sequencing

Total RNA was extracted from Asian sea bass fish with Trizol (GeneALL, Korea) and the first chain complementary deoxyribonucleic acid (cDNA) was synthesized according to the manufacturer’s instructions (Thermo, Life Sciences). Dicentracin-like coding gene **(**XM_018688317.1**)** was amplified using the specific forward (5- ATA GGA TCCATGAAGTGTGTTATGCTTTTTC -3) and reverse (5- ATA CTC GAGTCAAAATGAGGCCTGATAATC-3) primers that were designed using Gene Runner software on the basis of dicentracin-like gene sequences(XM_018688317.1). After amplification of the gene, PCR product was extracted from agarose gel and digested with BamH1/Xho1 enzymes, and inserted in BamH1/Xho1 sites in the pET28b vector. *E*.*coliDH5α* cells were transformed ligated plasmid and colony containing recombinant plasmid was selected by the colony PCR method using T7 universal primers. Then the recombinant plasmid was extracted and the inserted DNA was sequenced (Source: BioScience, UK).

### Semi-quantitative RT-PCR

Total RNA was extracted from the skin, the head kidney, the lung and the intestine tissue (100 mg) using RiboEx Total RNA (GeneALL, Korea). The first chain of complementary deoxyribonucleic acid (cDNA) was synthesized according to the manufacturer’s instructions (Thermo, Life Sciences); and was used as a template to amplify dicentracin-like coding gene (XM_018688317.1) by a polymerase chain reaction. The specific primers were designed according to the sequence of the dicentracin-like gene (XM_018688317.1) and was amplified using the following primers, including the forward primer: 5- ATGAAGTGTGTTATGCTTTTTC -3 and reverse primer: 5- TCAAAATGAGGCCTGATAATC -3. For semi-quantitative RT-PCR, EF1α gene was used as an internal control; the forward and reverse primers for EF1α gene were 5’-TGCTGATTGTGGCTGCTGGTACTG -3’ and 5’- GGTGTAGGCCAGCAGGGCGTG-3, respectively. The dicentracin-like gene expression was relatively quantified with Image software. The relative quantification was determined by the expression level of dicentracin-like gene relative to the EF1α gene expression in each tissue.

### Peptide synthesis

Dicentracin-like and morocidin peptides were synthesized by the solid-phase synthesis method using N-(9-fluorenyl) methoxycarbonyl (Fmoc) chemistry by Pepmic (Suzhou, China) and were purified with RP-HPLC, using the SHIMADZU Inertsil ODS-SP(4.6*250 mm* 5μm) column. Peptides were eluted by a 0–100% H2O/acetonitrile gradient, with 0.1% trifluoroacetic acid for 30 minutes. The homogeneity of peptides were estimated with analytical high-performance using an Inertsil ODS-SP (4.6*250 mm* 5μm) column that showed 95% purity. The correct atomic masses of purified peptides were verified with mass spectrometry (MS). The N-and C-terminals of peptides were modified with 5-FAM and an amine group (NH2, amidated C-terminus), respectively.

### Retention time of peptides

The retention time of the peptides were calculated by RP HPLC using an analytical SHIMADZU Inertsil ODS-SP (4.6*250 mm* 5μm) column. A linear gradient of 0–100% acetonitrile supplemented with 0.1% trifluoroacetic acid was used to elute peptides at a flow rate 1ml/minute for 30 minutes. The highest peak in 214 nm was considered as the retention time for each peptide.

### Minimum inhibitory concentration (MIC)

The antimicrobial activity of dicentracin-like peptide and moronecidin against the gram-negative bacteria[*Escherichia coli* (ATCC 25922), *Pseudomonas aeruginosa*(ATCC 10662)and *Acinetobacterbaumannii* (clinical isolate)], gram-positive bacteria [*Staphylococcus aureus* (ATCC 25923), *Staphylococcus epidermidis*(ATCC 1435) and *Staphylococcus aureus* (MRSA)] and yeast clinical isolate (*Candida glabrata*, *Candida tropicalis*, *Candida albicans*) was assessed according to the broth microdilution technique as described by a nature protocol for MIC evaluation of antimicrobial peptides [21]. Briefly, a series of two-fold dilutions of each peptide(300, 150, 75, 37.5, 18.75, 9.37, 4.68 and 2.34μg/ml) was created with 0.01% acetic acid and 0.2% BSA (bovine serum albumin) and then 50 μl of each peptide dilution was added to each well of polystyrene flat bottom 96- well microplate (Sigma-Aldrich, Germany). Microbial suspension was diluted with BHI medium or RPMI medium to provide density of 1×10^6^ cell/ml and 50 μl of diluted microbial suspension was added to wells containing 50 μl of peptide solution, providing a final concentration of 5×10^5^ cell/ml and incubated for 18 hours at 37 °C. The sterility control well contained 100 μl of BHI (or RPMI), while the growth control well contained 100 μl of microbial suspension (5×10^5^ cell/ml). The MIC values was defined as the lowest peptide concentration that prevented visible growth of the bacteria. Three independent tests were performed for each peptide concentration.

In the next step, the pH and salt concentration effect on MIC of AMPs were assayed. To evaluate the effect of cations on the antibacterial activity of AMPs, bacteria were cultured in the BHI medium containing increased concentrations of monovalent or divalent salt including NaCl (62,125,250 and 500 mM), MgCl_2_ (1,5, 10 and 30 mM), respectively.

The antibacterial activity of both peptides were assessed against *Enterococcus faecalis*, a bacteria resistance to alkaline environments (ATCC:6057), at different pH values (pH range: 7.4, 8.5, 9.5, or 10.5) [22]. The bacteria were cultured in the BHI medium adjusted to varying pH values (pH range: 7.4, 8.5, 9.5, and 10.5). After incubation at 37°C for 18 hours, the MIC values were determined.

### Time-kill analysis

Time-Kill assay was performed to investigate bactericidal activity of both peptides against antibiotic resistant bacteria including *S*. *aureus* (MRSA), and clinical *A*. *baumannii* isolate. For this purpose, overnight culture of bacteria were diluted with BHI in the wells of 96-well microplate (Sigma-Aldrich, Germany) to give final density of 5×10^5^ cell/ml in the presence of each peptide at concentrations of 2×, 1× and 1/2 × MIC. Colony count was performed after incubation at 37°C for 0, 2, 4, 8, 12 and 24 h by plating of 10-fold dilutions on LB agar. Minimum bactericidal concentration (MBC) was considered as the lowest concentration of antimicrobial that caused at least a 99.9% (equal to a ≥3-log10 reduction) decrease in the initial inoculum. The lower limit of detection for time-kill assays was 2 log10 CFU/ml.

### Binding affinity of AMPs to the gram-negative and the gram-positive bacteria

Antimicrobial peptides bind to the bacterial cell wall through an electrostatic interaction between the positive charge of AMPs with the negative charge of the bacterial cell wall [23]. We evaluated the binding affinity of dicentracin-like and moronecidin to gram-negative and gram-positive bacteria using a cell-based fluorometric ELISA. To determine optimal binding conditions, different concentrations of the bacteria (12.5×10^7^, 25×10^7^, 5×10^8^, 1×10^9^ cell/ml) and 5-FAM-labeled peptide (1.25, 2.5, 5, and 10 μg/ml) were prepared. Bacterial suspension from each bacterial concentration was added to 4 wells of Maxisorp 96-well microplates (each well 100 μl) and incubated for 3 hours at 37°C without agitation. The planktonic cells were carefully discarded by pipetting and the adherent cells were washed three times with PBS buffer (pH 7.4). Bacteria were fixed with 100 μl of 4% paraformaldehyde and incubated for 0.5 hours at 37°C. The paraformaldehyde was removed and the wells were washed three times with PBS buffer (pH 7.4). The wells were air-dried and fixed cells were confirmed with an inverted microscope. Subsequently the wells were blocked with 200 μL/well of 5% (w/v) skimmed milk or 2% bovine serum albumin at 37°C for 3 hours and were washed three times with PBS-T buffer (0.05% Tween-20 in PBS); then 4 × 100 μl of each 5-FAM-labeled peptide concentration (1.25, 2.5, 5, and 10 μg/ml) were added to 4 wells containing bacterial dilution of each concentration (12.5×10^7^, 25×10^7^, 5×10^8^, 1×10^9^ cell/ml) and incubated for 2 hours at 37°C (plate was covered during incubation). After three washing steps with PBS-T buffer, the fluorescence intensity was measured using a fluorescence microplate reader (BioTek Synergy 4, USA) at 490/560 nm (Ex/Em). Cell-free wells blocked with 5% (w/v) skimmed milk or 2% BSA and treated with 5-FAM-labeled AMP were used as blank wells. The optimal conditions for evaluating binding affinity of AMPs to bacteria were determined as follow: 12.5×106 cell/well, 0.25 μg/well peptide and 2% BSA (for blocking). Under the optimized conditions, the binding affinity of both peptides to bacteria was assessed.

### Evaluation of the pH effect on binding affinity of AMPs to bacteria

The binding affinity of the peptides to the cell wall of *E*. *faecalis* was evaluated in PBS adjusted to different pH values (pH range: 7.4, 8.5, 9.5, or 10.5) by a cell-based fluorometric ELISA test. Like the previous step, bacteria were fixed on the well surface of microplate, blocked and washed. The 5-FAM-labeled AMPs were diluted in PBS buffers adjusted to varying pH values (pH range: 7.4, 8.5, 9.5, or 10.5) to give a final concentration of 2.5μg/ml; then 100μl of diluted peptide was added to cell-fixed well (peptide, 0.25 μg/well) and incubated for 2 hours at 37 °C. The wells were washed three times with PBS-T buffer. The fluorescence intensity were measured using a fluorescence microplate reader (BioTek Synergy 4, USA) at 490/560 nm (Ex/Em). The wells without fixed cell that were only blocked with 2% BSA were incubated with peptide and were used as blank wells.

### Evaluation of the salt effect on binding affinity of AMP to bacteria

Considering potential impact of salt on antimicrobial activity of AMPs due to their interference with the electrostatic interaction between AMPs and the bacterial cell wall [24, 25], investigation of the salt effect on the binding affinity to bacteria appears to be of importance for antimicrobial characterization of a given AMP. As described for analysis of pH effect, bacteria were fixed on the well surface of microplate, blocked and washed. The peptide solution was diluted with two-fold serial dilutions of monovalent (62, 125, 250, and 500 mMNaCl) or divalent salt (1, 5, 10, and 30 mM MgCl_2_) to provide a final concentration of 2.5μg/ml of peptide. 100-μl of diluted peptides (2.5μg/ml) was added to cell-fixed well, and then microplate was incubatedat 37°C for 2 hours. After three washing steps with PBS-T, the fluorescence intensity was measured using a fluorescence microplate reader (BioTek Synergy 4, USA) at 490/560 nm (Ex/Em). The wells without fixed cell that were only blocked with 2% BSA were incubated with peptide and were used as blank wells.

### Assessment of antiadhesive and antibiofilm activities of AMPs

Surface attachment is the essential first step in effective colonization or biofilm formation of bacteria [26]. Existence of some certain factors such as teichoic acids and lipopolysaccharide appear to be crucial in this function [27]. Given the importance of surface attachment and biofilm formation, antiadhesive and antibiofilm activities of the peptides against *S*.*aureus* were evaluated, as described elsewhere [24, 25]. Protocols for evaluating antiadhesive and antibiofilm potency are similar; however the antibiofilm assay needs to incubate bacteria in the presence of AMPs for a longer time (24 hours), compared with the antiadhesive assay (1hour). Overnight cultures of bacteria were diluted with BHI to provide OD600 = 0.05 and then 1:100 suspended bacteria were added to wells containing BHI and the peptides. Overnight *S*. *aureus* suspensions were incubated in the presence of the peptides at subinhibitory concentrations of 1/2xMIC to 1/32xMIC for 1hour (antiadhesive assay) or 24 hours (antibiofilm assay) at 37°C without shaking to allow bacterial binding. After incubation, the planktonic cells were carefully aspirated from wells, and then wells were washed three times with PBS (pH value = 7.4). Fixing the attached cells by 200 μl of 4% paraformaldehyde and incubating microplate for 30 minutes at 37°C were followed by the subsequent steps including removing paraformaldehyde, air-drying microplate, staining the fixed cells for 2 minutes (with 200 μl of 0.41% of crystal violet), removing the extra dye, washing wells with 200μl PBS three times, solubilizing crystal violet by 95% ethanol and shaking microplate for 10 minutes. A 100-μl of solubilized crystal violet solution was transferred to wells of a new microplate and its absorbance was measured at 595 nm using a microplate reader. The well without peptide was used as binding control. The alkaline pH effect on antiadhesive and antibiofilm activities of AMPs against *E*. *faecalis* were assayed. For this purpose, bacteria were cultured in the BHI medium adjusted to varying pH values (pH range: 7.4, 8.5, 9.5, and 10.5) in the presence of the peptides at subinhibitory concentration of 1/32×MIC. Then, antiadhesive and antibiofilm assay was performed as described above.

### Hemolytic activity assay

The hemolytic activity of dicentracin-like peptide and moronecidinpeptide was assayed with human red blood cells (hRBCs) as described elsewhere [28]. After washing human RBCs with PBS, PBS was added to the pelleted RBCs to generate a RBCs suspension with a concentration of 4% v/v. Hemolytic activities of peptides were measured in concentrations of 75, 37.5, 18.75, 9.37, 4.68, 2.34 and 1.17μg/ml. For this purpose, mixtures containing RBCs suspension (100μl) and peptide dilutions (100μl) were incubated at 37 °C for 1 hour; then, RBCs were collected by centrifugation at 1000 g for 10 minutes. The supernatants of each dilution was divided into wells (100 μl/well) in a 96-well plate and the absorbance was read at 405 nm with the microplate reader (BioTek Synergy 4, USA). The triton X-100 (0.1%) and the RBCs suspension were used as positive and negative hemolysis control, respectively. The percentage of hemolytic activity of AMPs was measured according to the following equation [29]:

$$\left[\left({\text{A}}_{\text{peptide}}{\text{-A}}_{\text{PBS}}\right)/\;\left({\text{A}}_{0.1\%\;\text{triton}\;\text{X-}100-}{\text{A}}_{\text{PBS}}\right)\right]\;\times 100$$

In addition, the peptide concentration, with 50% hemolytic activity against human red blood cells (HC50), was estimated for both AMPs.

### Statistical analysis

Data analysis was performed using the Prism software (Version 6; GraphPad).

ANOVA test was used to analyse data and P values <0.05 were statistically considered significant.

## Results

### Database searching

When the signal peptide of moronecidin (AMP from *Moronesaxatilis*) was used as a query in BLASTP against the Asian sea bass, only a putative novel AMP precursor with the name of dicentracin-like peptide (XM_018688317.1) was found. The amino acid sequence of this AMP was used in BLASTP and it was revealed that its sequence is not very similar to other identified AMPs. Piscidin-4 and -5 precursors from *M*. *chrysops* and *saxatilis hybrid* (GenBank ADP37959.1 and ADP37960.1), however, showed relative similarity with dicentracin-like peptide (XM_018688317.1).

### Cloning, sequencing, and sequence analysis of dicentracin-like

Following cDNA synthesis, the dicentracin-like coding sequence was inserted into pET28b vector and the recombinant plasmid pET28a was sequenced (S1 Fig). To define the motifs of the deduced dicentracin-like, its amino acid sequence was submitted in the motif finder, by which the two motifs including pleurocidin family (PF08107) and the testis-expressed sequence (PF15326) were determined (S1 Fig). A 22-amino-acid mature AMP was defined which had general properties of AMPs including cationic (total charge +6) and hydrophobic properties (0.321%) [30]. Piscidin-4 (*Epinepheluscoioides*) [31] and cathelicidin (from *Sarcophilusharrisii*) were found to have the most similarity to dicentracin-like peptide, with a 52% similarity [32]. The alignment of dicentracin-like peptide with other known piscine AMPs revealed a high identity in the signal peptide sequence, but a high variability in the mature region of both AMPs (S2 Fig). Schiffer–Edmundson helical wheel modeling and secondary structure prediction demonstrated that the hydrophobic and the hydrophilic residues are located on opposite sides in the alpha-helix structures of dicentracin-like and moronecidin, and these AMPs have amphipathic alpha-helix conformations (Fig 1).

An assessment of physicochemical properties of dicentracin-like and moronecidin indicated that dicentracin-like has more positive charge and higher water solubility, compared with moronecidin. Moronecidin possesses more hydrophobic properties than dicentracin-like, while both peptides have similar amphipathic properties (Table 1).

### Tissue-specific expression of dicentracin-like peptide

The expression of dicentracin-like peptide was analyzed in fish tissues including the head kidney, the skin, the intestine, and the gill by semi-quantitative RT-PCR. The highest expression was observed in the head kidney. The relative expression level of dicentracin-like (relative to that of EF1α gene as an internal control) in the head kidney, the skin, the gill, and the intestine were 1.25-, 0.30-, 1-, 0.89-, and 0.49-fold (S3 Fig).

### Retention time of peptides

The evaluation of RP HPLC retention time revealed that moronecidin was eluted with two peaks including a tiny peak in 18.65 minute and the main peak in 21.58 minute, while dicentracin-like was eluted only with one peak in 20.518 minute (Table 1).

### Antimicrobial activity

**Fig 2:**
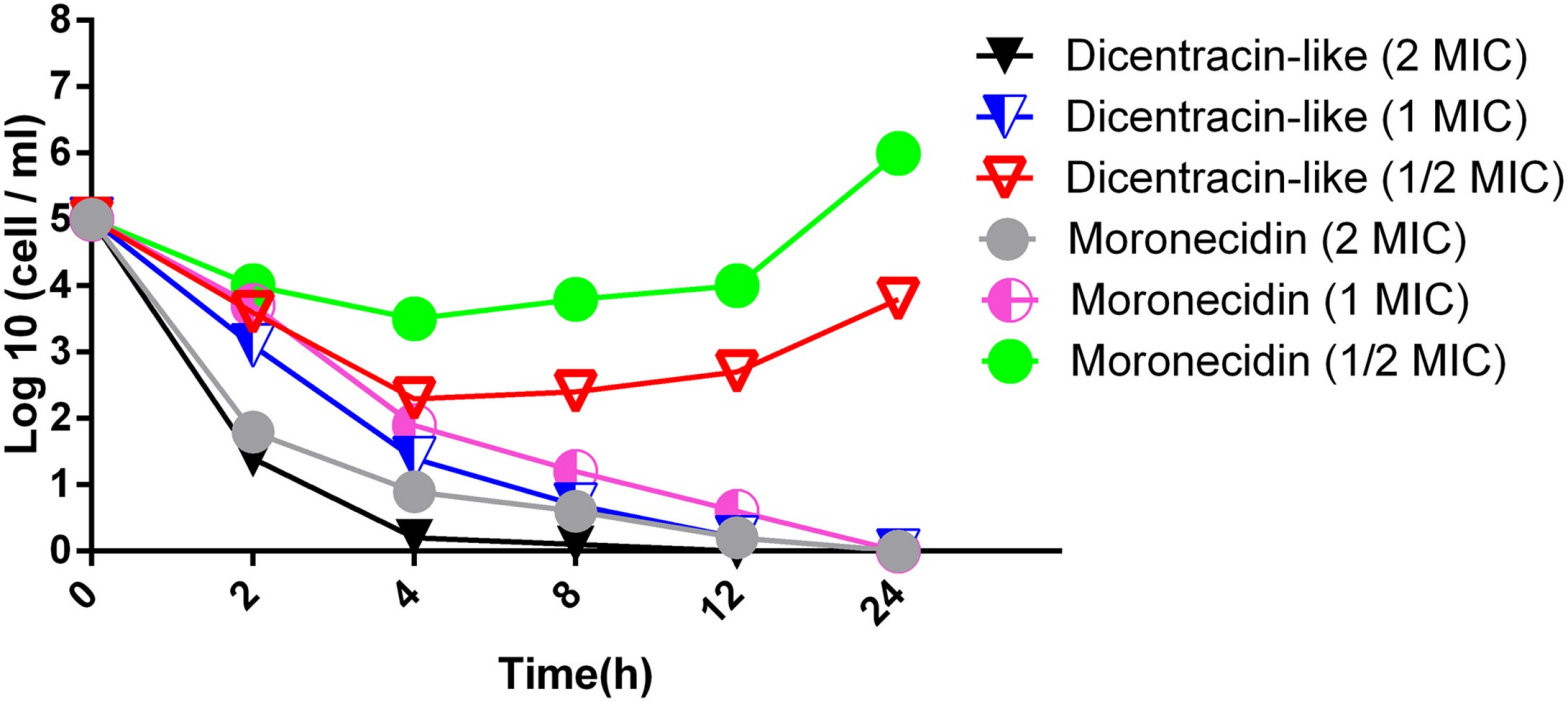
Effects of dicentracin-like and moronecidin on the viability of *A*. *baumannii* (clinical isolate). All data represent mean of two independent experiments.

The antimicrobial activity of dicentracin-like peptide and moronecidin was evaluated against the gram-negative bacteria [*E*. *coli* (ATCC 25922), *A*.*baumannii* (clinical isolate), *P*. *aeruginosa* (ATCC10662)], the gram-positive bacteria [*S*. *aureus* (ATCC 25923), *S*. *epidermidis*(ATCC 1435)] and yeast clinical isolate (*C*.*glabrata*, *C*.*tropicalis*, *C*. *albicans*). The results showed that both AMPs have high activity against gram-positive bacteria (*S*.*epidermidis*, *S*.*aureus*) and *E*.*coli*, while they showed low activity against other gram-negative bacteria including *P*. aeruginosa and *A*.*baumannii* clinical isolate. *S*. *epidermidis* showed more sensitivity, while *P*. *aeruginosa* showed the lowest sensitivity to both peptides. The dicentracin-like peptide showed higher activity than moronecidin against all bacteria (except against *S*.*aureus*), whereas moronecidin was more potent than dicentracin-like peptide against the standard *S*. *aureus* strain and *S*. *aureus* (MRSA) (Table 2). Both peptides exhibited effective activity against *C*. *albicans* and *C*.*tropicalis*, but not against *C*.*glabrata*. The results of time-kill assay against clinical *A*.*baumannii* isolate demonstrated that bactericidal activity of dicentracin-like and moronecidin was relatively similar at suprainhibitory and inhibitory concentration of 2X and 1XMIC, however the dicentracin-like peptide displayed more potent activity than moronecidin at subinhibitory concentration of 1/2XMIC. Bactericidal activity of dicentracin-like and moronecidin was observed at concentration of 2X and 1XMIC after 2 and 4 hours of incubation, respectively (Fig 2).

**Table 2:** The minimum inhibitory concentration (MIC) of dicentracin-like and moronecidin.

Bacteria strains	Culture broth	Antimicrobial peptides((μg/ml)	
Gram-negative bacteria		Dicentracin-like	Moronecidin
*Escherichia coli* (ATCC 25922)	Brain heart infusion	4.68	9.37
*Pseudomonas aeruginosa* (ATCC10662)	Brain heart infusion	37.5	75
*Acinetobacterbaumannii*(Clinical isolate)	Brain heart infusion	18.75	37.5
**Gram-positive bacteria**			
*Staphylococcus aureus*(ATCC 25923)	Brain heart infusion	4.68	2.34
*Staphylococcus epidermidis* (ATCC 1435)	Brain heart infusion	1.17	2.34
*Staphylococcus aureus***(MRSA)**	Brain heart infusion	4.68	2.34
**Yeast**			
***Candida albicans*** (clinical isolate)	RPMI medium	4.68–9.37	9.37
*Candida tropicalis*(clinical isolate)	RPMI medium	4.68–9.37	9.37
*Candida glabrata*(clinical isolate)	RPMI medium	>150	>150

Salt can reduce antimicrobial activity of AMPs by disruption of the electrostatic interaction between AMPs and bacterial cell wall. AMPs should be able to tolerate physiological salt concentrations to become effective in the clinical condition [33].

**Fig 3:**
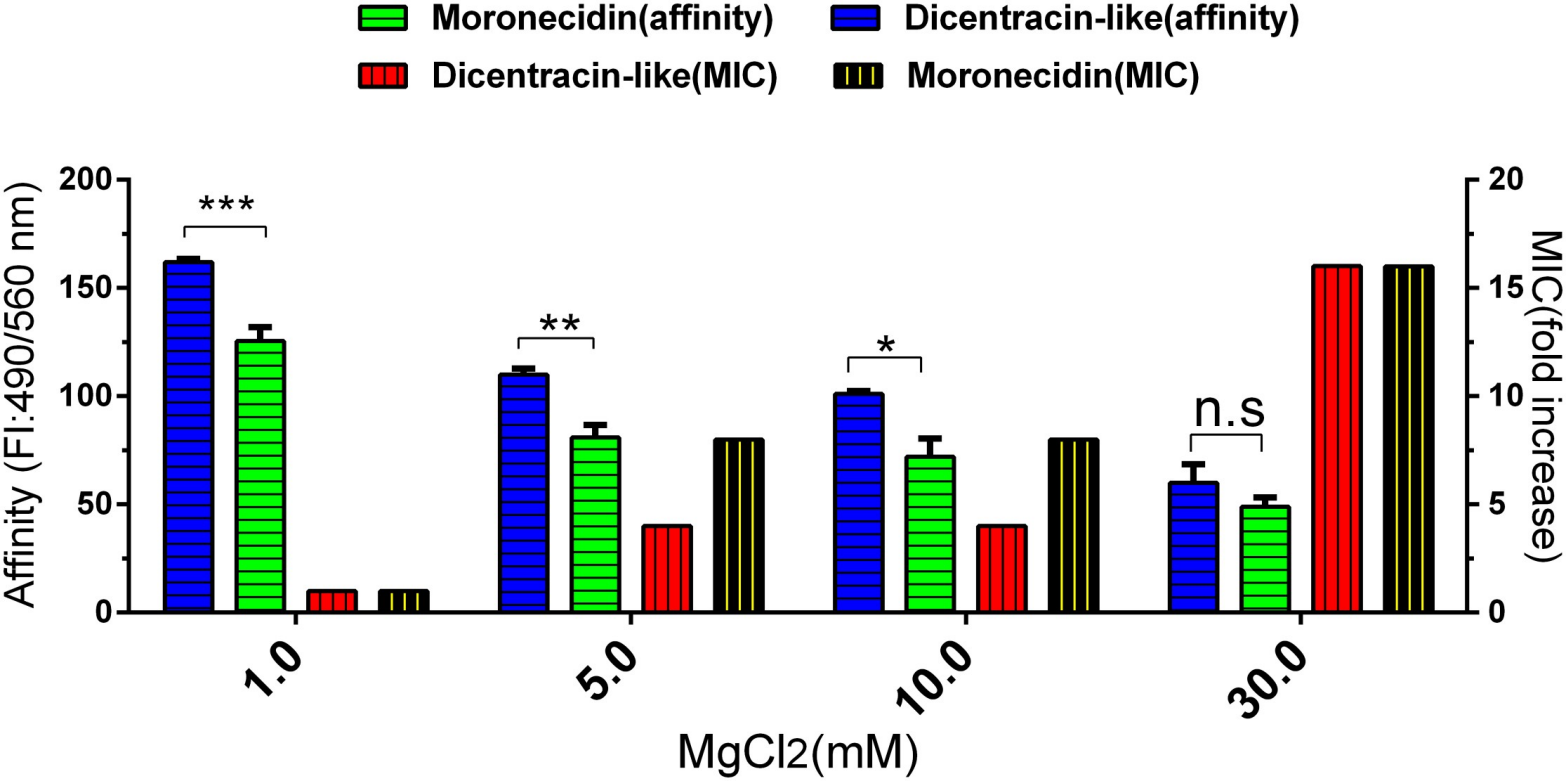
Evaluation of various MgCl2 concentration on the MIC and AMPs binding to bacteria. Binding affinity of moronecidin and dicentracin-like to bacteria (E.coli) were measured with a cell-based fluorometric ELISA. Fluorescence intensity (FI) of wells were measured in various MgCl_2_ concentrations (1, 5, 10 and 30 mM) on a microplate reader at excitation /emission (Ex/Em) = 490/560 nm. Fold increase scores in MIC were calculated by MIC _(MgCl2)_ /MIC. The scores represent three independent experiments.*P≤0.05, **P≤0.02, ***P≤0.01.

Therefore, we evaluated the effect of various salt concentrations on the MIC of both peptides against *E*.*coli*. Compared to monovalent salt (NaCl), divalent salt (MgCl_2_) possessed more antagonistic effect on antibacterial activity of peptides. Increasing concentration of the salt reduced the antimicrobial activities of both peptides, while any reduction in the activity of both peptides was not observed in physiological salt concentrations. In the presence of 5 mM and 10 mM MgCl_2_, the MIC value of the dicentracin-like and moronecidin peptides increased by 4- and 8-fold, respectively (Fig 3 and Table 3), whereas the MIC value of dicentracin-like and moronecidin peptides increased by 2-fold in the presence of 500 mM NaCl. Furthermore, the MIC of both peptides increased by 16-fold in the presence of 30 mM MgCl_2_ (Table 3).

**Table 3:** Activity (MIC) of AMPs against *E*.*coli* in various salt concentrations.

	Dicentracin-like(μg/ml)	Moronecidin(μg/ml)
**NaCl(mM)**		
62	4.68	9.37
125	4.68	9.37
250	6.05	13.53
500	9.37	18.75
**MgCl_2_(mM)**		
1	4.68	9.37
5	18.75	75
10	18.75	75
30	75	150
**Physiological** **salt concentrations** **(150 mM NaCl** **3 mM CaCl**_**2**_ **2 mM MgCl**_**2**_)	4.68	9.37

**Fig 4:**
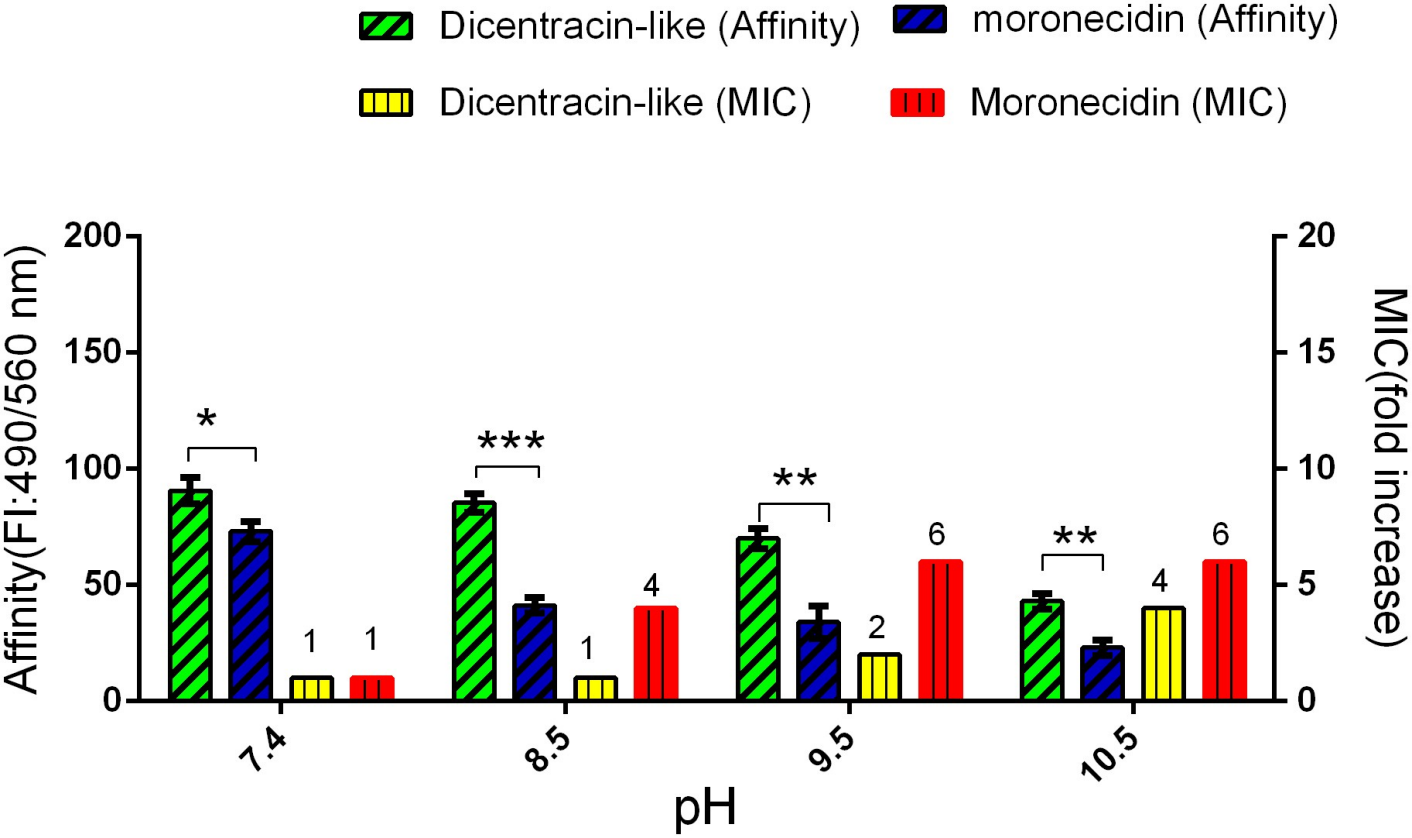
Evaluation of the pH effect on the AMPs binding affinity to *E*. *faecalis* and their MIC. Binding affinity of moronecidin and dicentracin-like to bacteria was measured with a cell based fluorimetric ELISA. Fluorescence intensity of wells were measured on a microplate reader at excitation /emission (Ex/Em) = 490/560 nm. Data represent mean ± SD for three independent experiments. *P _=_ 0.01, **P = 0.002, ***P≤0.0001.

Evaluation of the effect of alkaline pH on antibacterial activity demonstrated that the increasing of pH value reduced antibacterial activity of both peptides, especially for moronecidin (Fig 4). The increasing of pH to 8.5 was caused a rapid decrease (4-fold) in antibacterial activity of moronecidin, whereas no reduction in antibacterial activity of dicentracin-like was observed. Overall, dicentracine-like was more potent than moronecidin against *E*. *faecalis* (ATCC:6057) at alkaline pH value.

### Binding affinity of AMPs to bacteria at different pH values

The pH value of healthy skin, and chronic and acute wounds are different. The natural skin surface is slightly acidic(pH value = 4.2–5.6), but infected wounds are alkaline (pH value = 6–10) [26, 27]. Here, we evaluated the effect of different pH values on the electrostatic interaction of the AMPs (moronecidin and dicentracin-like) against *E*. *faecalis* [25]. The obtained results demonstrated that the increasing of pH value to 8.5 leads to a rapid decrease in affinity binding of moronecidin to bacteria. In addition, dicentracin-like has significantly higher binding affinity to bacteria, especially in pH value of 8.5, compared to moronecidin (Fig 4).

### Binding ability of AMPs to gram-negative and gram-positive bacteria

**Fig 5:**
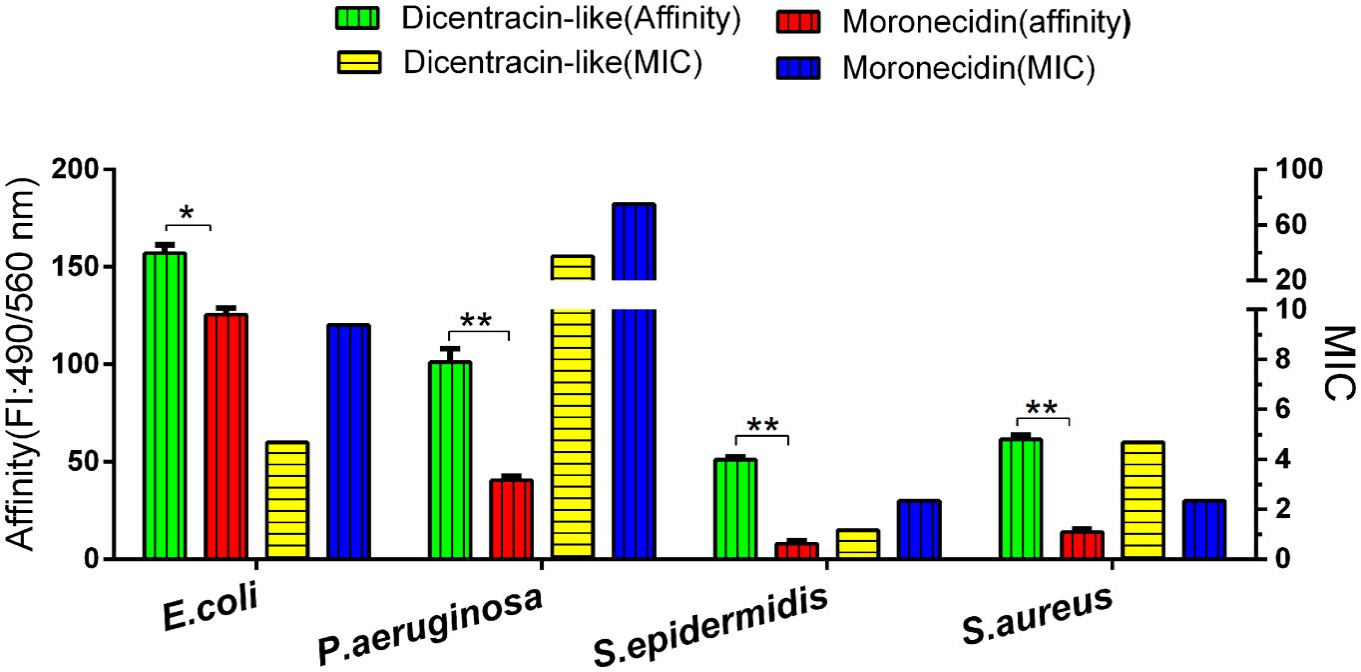
Comparison of the binding affinity of AMPs to gram-negative bacteria and gram-positive ones and their MICs (μg/ml). Binding affinity of moronecidin and dicentracin-like to bacteria was measured with a cell-based fluorometric ELISA. Fluorescence intensity (FI) of wells was measured on a microplate reader at excitation /emission (Ex/Em) = 490/560 nm. Binding affinity scores represent mean ±SD of three independent experiments. *: *p* ≤ 0.01, **: *p* ≤ 0.001, and ns: *p ≥ 0*.*05*.

The results of binding ability assay of AMPs to gram-negative and gram-positive bacteria demonstrated that both peptides possess higher binding ability to gram-negative bacteria, compared with gram-positive ones, especially for *S*.*epidermidis* (Fig 5). Dicentracin-like showed higher binding affinity to bacteria than moronecidin.

### Effect of salt concentration on AMPs binding to bacteria

Electrostatic interactions between AMPs and bacteria cell wall which is the prerequisite for antibacterial activity can be hindered by salt [24, 34]. We evaluated the binding of AMPs to bacteria cell at various salt concentrations. Results showed that the increasing concentration of monovalent cation (NaCl) decrease the binding affinity of moronecidin and dicentracin-like peptides to bacteria. Monovalent cation (NaCl) equally reduced the binding affinity of moronecidin and dicentracin-like peptide to bacteria, while 1.6 and 1.74-fold reduction were observed in affinity binding of dicentracin-like and moronecidin peptides to bacteria, respectively, in the presence of 10 mM MgCl_2_concentration(Fig 3). Like antibacterial activity, dicentracin-like exhibited greater affinity binding compared with moronecidin, in the presence of 10 mM MgCl2. No significant difference was observed in affinity binding of peptides in the presence of 30 mM MgCl2.

### Inhibitory effect of AMPs on surface attachment and biofilm formation

**Fig 6:**
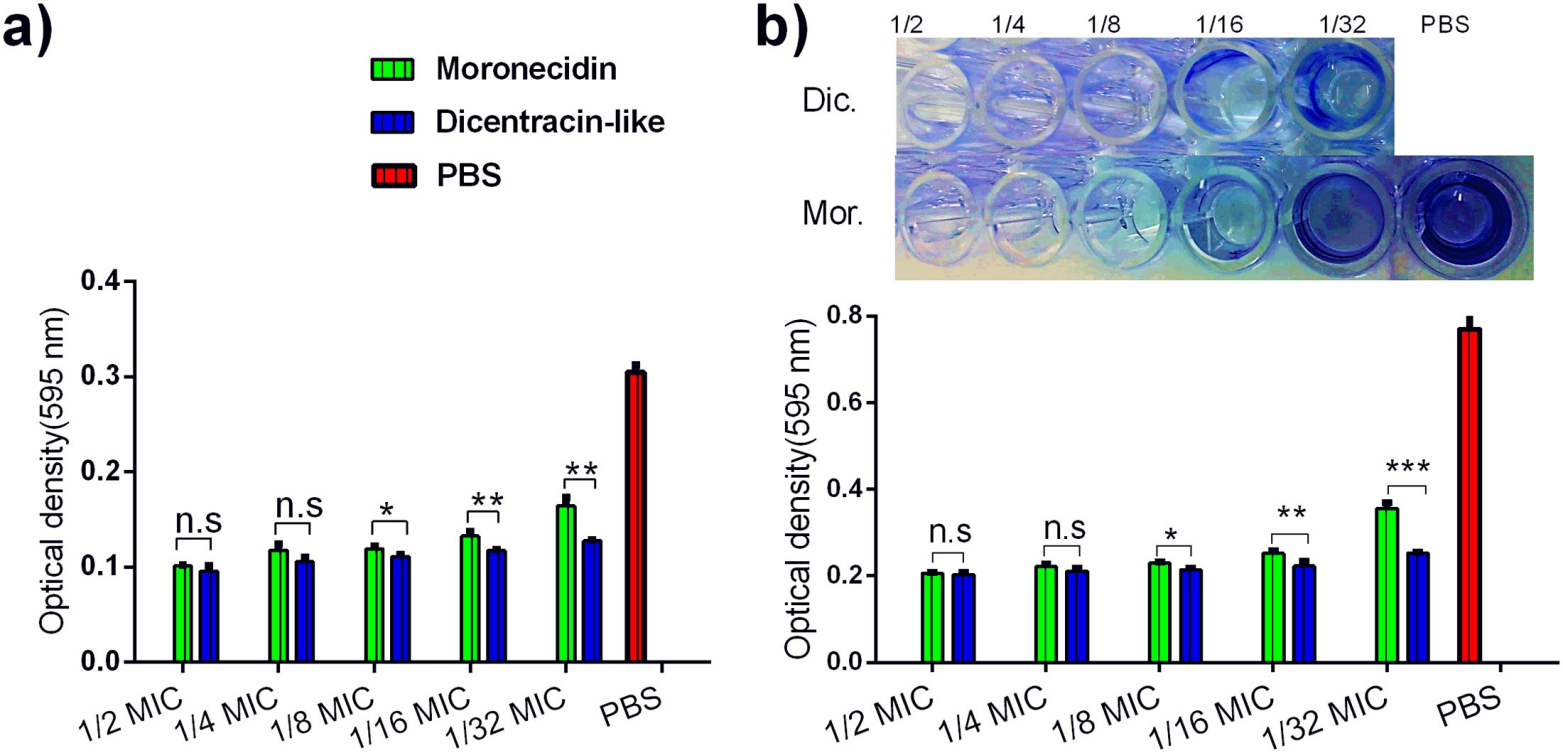
Inhibition of well-surface attachment and biofilm formation of S.aureus by moronecidin and dicentracin-like peptide. Inhibition of surface attachment (a) and biofilm formation was investigated at two-fold serial dilutions of peptides (1/2 to 1/32 MIC). The number of attached cells were measured by ELISA microplate reader and represented as mean (±SD) of three independent experiments. PBS represent well without peptide treatment (negative control)*, *p*-*value* ≤ 0.05; ** *p*-*value* ≤ 0.01; *** *p*-*value* ≤ 0.000.1; n.s, no significant; *p*-*value≥ 0*.*05* was considered as no significant difference.

**Fig 7:**
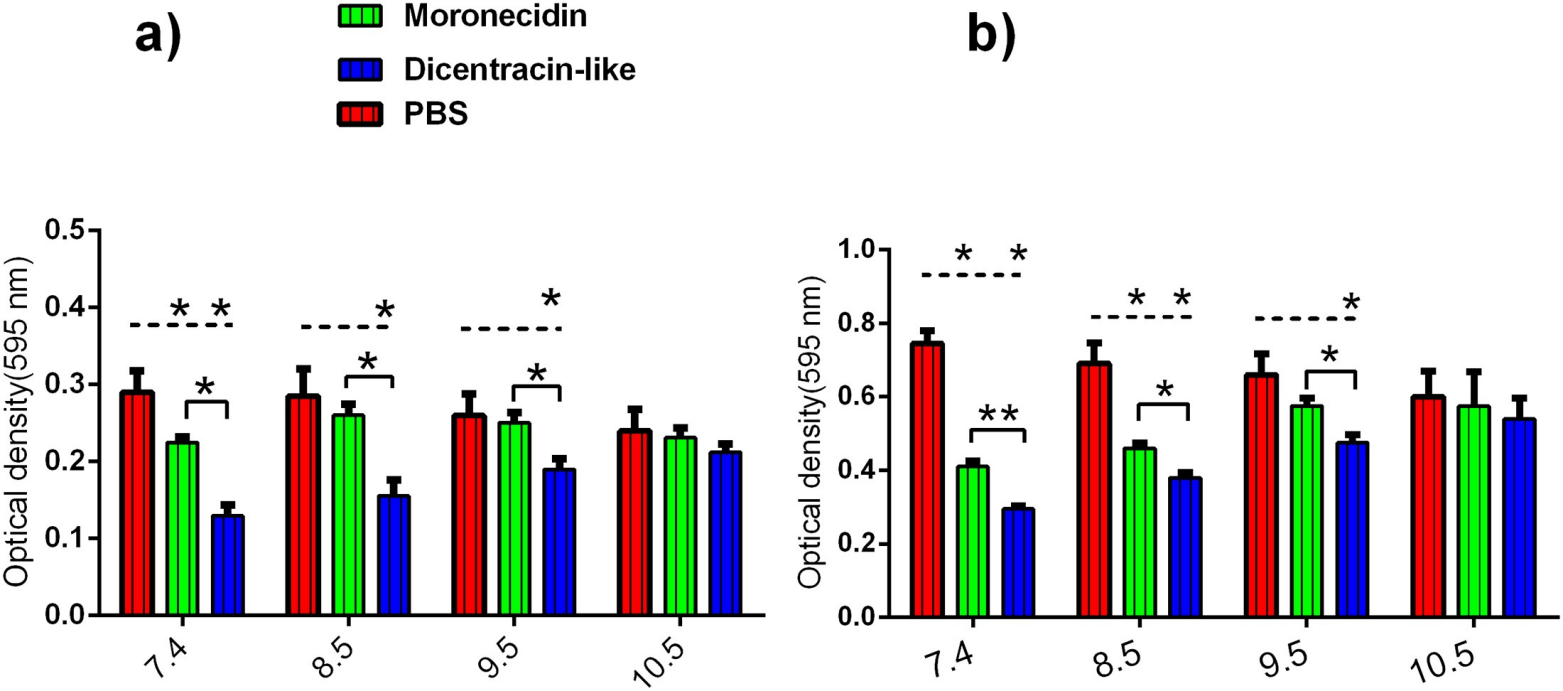
Evaluation of the pH effect on antiadhesive and antibiofilm activity of AMPs against *E*. *faecalis*. Inhibition of adhesion (a) and biofilm formation (b) was investigated in the presence of peptides at 1/32 × MIC.

The evaluation of the inhibition of bacterial attachment to inside well-surface by peptides revealed that wells containing higher concentration of peptides have less attached bacteria, compared with wells containing lower concentration of peptides. Antiadhesive and antibiofilm effects of both peptides were exerted in a dose-dependent manner, although greater effects were exhibited by disentracin-like, relative to moronecidin (Figs 6 and 7).

Furthermore, investigation of the pH effect demonstrated that moronecidin significantly decreases the bacterial adhesion at pH 7.4 and biofilm formation at pH values from 7.4 to 8.5, relative to corresponding control, while dicentracin-like is able to significantly decrease the bacterial adhesion and biofilm formation at pH values from 7.4 to 9.5 (Fig 7). In addition dicentracin-like has higher antiadhesion and antibiofilm activity than mornecidin at pH values from 7.4 to 9.5, while no statically statistically significant difference was observed between activity of both peptides at pH value of 10.5.

### Hemolytic assay

**Fig 8:**
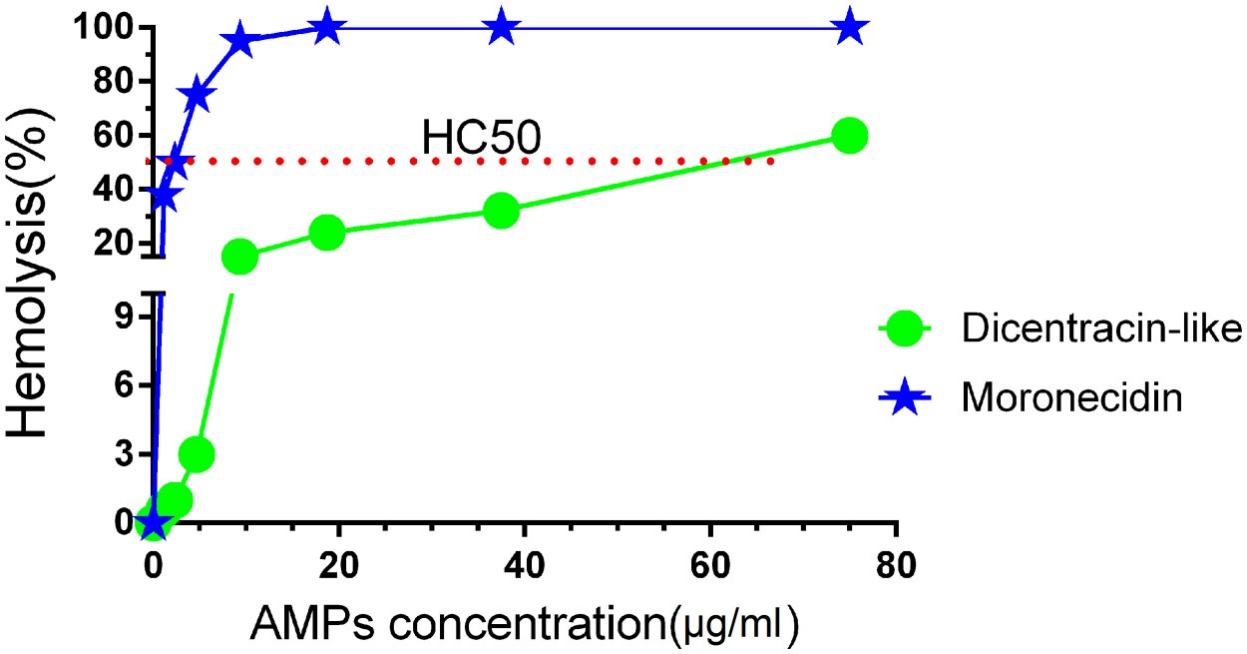
Hemolytic activity of peptides. Hemolysis of human RBCs by moronecidin anddicentracin-like against human RBCs was estimated at various concentration of AMPs. Red line represents HC50 for peptides.

Cytotoxicity of some AMPs is the main barrier to the clinical use of them; the hemolytic activity of the dicentracin-like peptide against human red blood cells (hRBCs) was examined in comparison to moronecidin. Moronecidin showed higherhemolyticactivity than dicentracin-like peptide, the HC_50_ for moronecidin and dicentracin-like were 57 μg/ml and 2.34 μg/ml, respectively (Fig 8). Dicentracin-like did not show100% hemolytic activity even in the peptide concentration of 75 μg/ml.

## Discussion

One of the most common health problems is the increasing resistance to conventional antibiotics among clinical microorganisms. To overcome this problem, researchers try to discover novel antimicrobial compounds lacking problems related to antibiotic resistance [30, 35, 36]. Antimicrobial peptides (AMPs) as a novel class of antibiotic agent have attracted the attention of researchers due to special antibacterial mechanism by which they directly disrupt the membrane structure. Given importance of cell membrane, the appearance of AMPs-resistance microorganism is very low [31, 32]. In the present study, the conserved signal peptide of moronecidin was used as a query in EST database against *Asian sea bass* (*Latescalcarifer*), resulting in the identification of a novel AMP, dicentracin-like. The amino acid sequence of mature dicentracin-like has very low similarity to other AMPs. Piscidin-4 (from *Epinepheluscoioides)* [37] and cathelicidin (from *Sarcophilusharrisii)* [38], with very low score similarity (52%), possessed the most similarity to dicentracin-like, so it was difficult to assign this peptide to any of the known classes of AMPs. In addition, the analysis of dicentracin-like gene expression in various tissues confirmed its expression in tissues involved in the immune system. Following the cloning and the sequencing of dicentracin-like, the mature dicentracin-like peptide and moronecidin were synthesized, followed by comparison of their antibacterial activity. Despite having broad-spectrum antimicrobial activity, both peptides exhibited more effective activity against gram-positive bacteria, relative to gram-negative ones. However, both peptides had a high-binding affinity to gram-negative bacteria, relative to gram-positive ones. A number of studies have revealed higher MICs of AMPs for gram-negative bacteria compared to gram-positive ones [39–41]. The outer membrane may be the reason behind the low antimicrobial activity of moronecidin and dicentracin-like against gram-negative bacteria, despite their high-binding affinity. LPS of the outer membrane of gram-negative bacteria has been particularly observed to have an active role in the function of AMPs [42–45]. Owing to the LPS barrier, there are fewer antibiotic candidates against multidrug resistant gram-negative strains [46, 47]. One of the mechanisms used by the LPS to decrease the efficacy of AMPs is induction of self-association of peptides [48–51] that may prevent the AMPs from passing through the cell wall to reach inner cell membrane [51–53]. On the other hand, since the surface negative charge of gram-negative bacteria is stronger than that of gram-positive ones [54], cationic AMPs may bind to gram-negative surfaces more easily. Compared to moronecidin, dicentracin-like showed higher activity against gram-negative bacteria, whereas moronecidin exhibited higher antibacterial activity than dicentracin-like peptide against *S*. *aureus*. The most likely reason for the difference in antibacterial activity against gram-negative bacteria between dicentracin-like and moronecidin may be related to their hydrophobicity. An analysis of data contained in the antimicrobial peptide database by Malanovic and Lohner revealed that the fraction of hydrophobic residues in the vast majority of AMPs is mostly between 30% and 50%, although it has been observed that AMPs being specific for gram-positive bacteria have a somewhat higher content of hydrophobic residues. However, it should be considered that the number of AMPs acting only against gram-positive bacteria is more than that against gram-negative bacteria. The above-mentioned data suggest that high hydrophobicity may prevent AMP from passing through the outer membrane and the cell wall to reach inner cell membrane [26]. Accordingly, since our results showed that the hydrophobicity of moronecidin is greater than that of dicentracin-like, the high hydrophobicity of moronecidin could explain its low activity against gram-negative bacteria, relative to dicentracin-like. Furthermore, in consistence with this finding, a previous study demonstrated, previous study demonstrated that the reduction of hydrophobic properties of non-polar face of moronecidin by the amino acid substitution on the non-polar face decreases its antibacterial activity against *S*. *aureus* [55]. In contrast, compared to moronecidin, dicentracin-like peptide exhibited more antiadhesive and anti-biofilm activity against *S*. *aureus*. Take together results indicated that a direct relationship between the binding affinity of peptides to bacteria and the inhibition of the surface attachment and biofilm formation, also the high antiadhesive and antibiofilm activities of dicentracin-like relative to moronecidin, may be related to its prominent positive charge and consequently its high-binding affinity to bacteria. Compared to moronecidin, dicentracin-like peptide showed more salt-resistant properties at salt concentration up to10mM, possibly because of its higher positive charge to compete with the positively charged ions (Mg2+) for binding to the bacterial cell wall. However, both peptides showed approximately equal reduction in antibacterial activity in the presence of 30 mM MgCl_2_, representing salt concentration at where the lowest difference was observed between bacteria- binding affinity score of both peptides. The positive charge of AMPs may be reduced in environments with alkaline pH such as the infected wounds [40]. The assessment of the binding affinity to bacteria and the antimicrobial and antibiofilm activity of AMPs under various pH values demonstrated that dicentracin-like peptide possesses higher affinity for bacteria, antimicrobial and antibiofilm potency than moronecidin at alkaline pH. This result is consistent with previous finding that the activity of histidine-rich antimicrobial peptides is dependent on an acidic environment. Furthermore, it demonstrated that replacement of histidine residue by arginine or lysine residue can increase their antibacterial activity at natural pH that this is because of very lower pka of side chain of histidine residue (imidazole groups, pKa = 6.0) at neutral pH value [56]. Hence, the high- binding affinity to bacteria, antimicrobial and antibiofilm potency of the dicentracin-like peptide under alkaline pH and high salt concentration of environment can be due to more arginine and less histidine residues compared to moronecidin. These properties allow dicentracin-like peptide to retain its bacteria-binding, antimicrobial and antibiofilm activity under alkaline pH of chronic and acute skin infection [26, 27]. The time-kill kinetic assay was performed to assess the bactericidal potency of dicentracin-like against *A*.*baumannii* (clinical isolate), which revealed that both peptides have rapid bactericidal activities against *A*. *baumannii* (clinical isolate).

**Fig 9:**
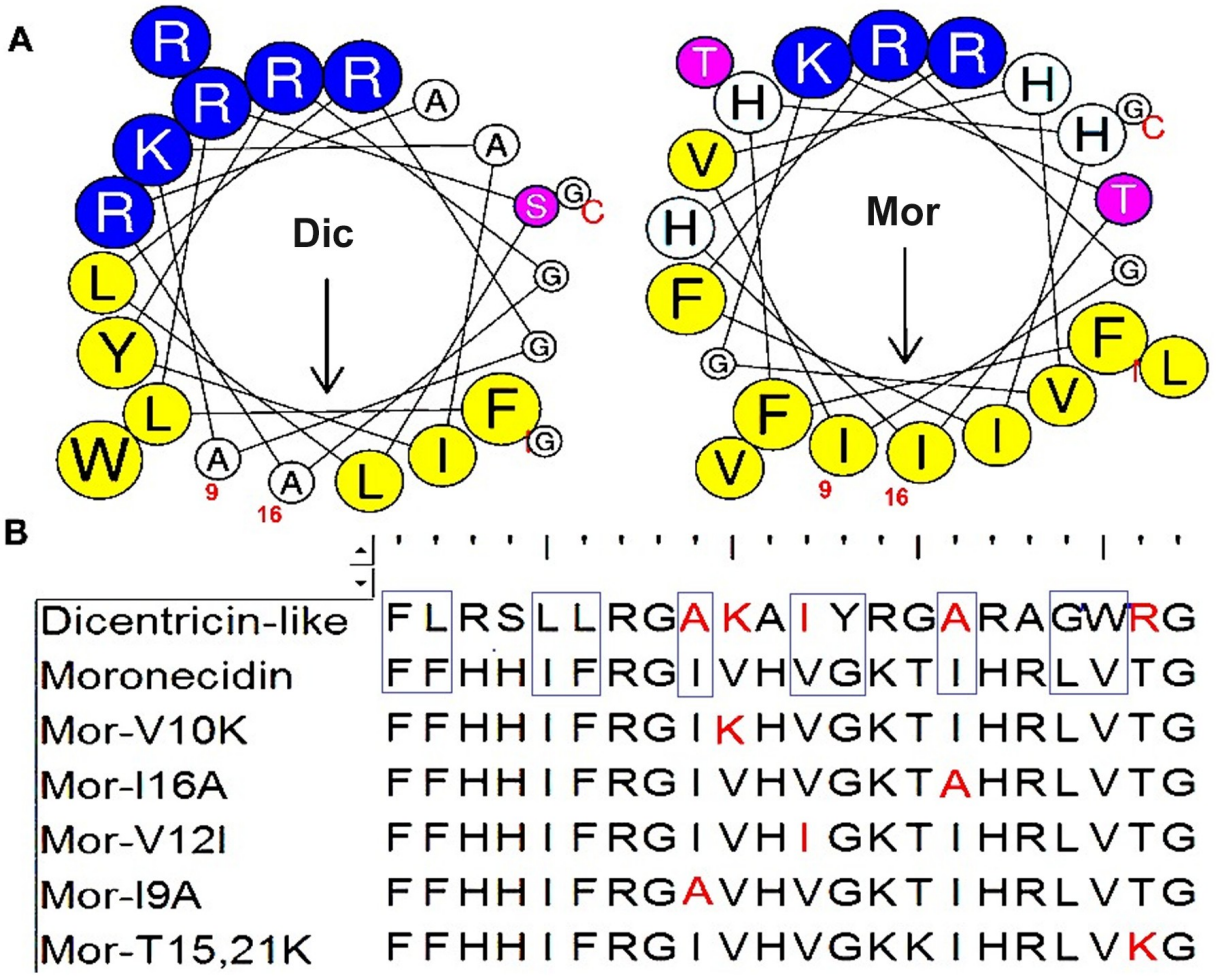
Helical wheel projections for dicentracin-like and moronecidin. Residues are numbered starting from the N-terminus. **B**: The alignment of dicentracin-like with moronecidin and its derivatives [57–59]. Red residues represent identical residues between dicentracin-like (Dic) and moronecidin (Mor) derivatives. Hydrophobic face residues in dicentracin-like and moronecidin are shown by rectangular.

Amit Kumar et al. studied the effects of amino acid substitutions on the antibacterial and cytotoxic activity of moronecidin. In that study, it was demonstrated that replacement of isoleucine 9 and 16 (I9 and 16) at the center of the non-polar face by alanine (I16A-moronecidin), remarkably reduced hemolytic activity against hRBC and retained the antibacterial activity, leading to a highest therapeutic index compared to other moronecidin analogs. In addition, replacement of valine12 at the border of the non-polar face (Val 12) by a more hydrophobic residue, isoleucine (V12I), considerably increased the antimicrobial activity and toxicity of moronecidin [57]. Surprisingly, like moronecidin analgos (I9A,16A and V12I), residues 9,16 and 12 indicentracin-like peptides arealanine (A9, 16) and isoleucine (I12), respectively (Fig 9).

The helical wheel projections revealed the integrity of the non-polar face on moronecidin, while the non-polar face of dicentracin-like peptide is broken by two low hydrophobic residues, alanine (Ala 9,16) that decrease hydrophobic property on non-polar face of moronecidin. It was demonstrated that substitution of residues at the center of the non-polar face by a hydrophobic residue (alanine) or a cationic residue (lysine, arginine) reduced hemolytic effect while retained antibacterial activity [57, 60–62]. Ina study conducted by Kumar *et al*, it was revealed that the increasing positive charge of moronecidin by the substitution of threonine(T) at position 15 and 21 by a cationic residue (moronecidin T15K, T21K) significantly reduced cytotoxicity while increased antibacterial activity [59]. Likemoronecidn T15K, T21K, the residue at position 21 of the dicentracin-like peptide is also a cationic residue (Fig 9).

These results in consistence with other studies [61, 63] confirm that the hemolytic activity of AMPs has a directed relation with retention time and the hydrophobic properties of non-polar face. In a study conducted by Son et al, it was demonstrated that all derivatives generated by Ala-scanning on the hydrophobic face, considerably exhibited decreased hemolytic activities [64].

A number of antimicrobial characteristics of dicentracin-like peptide were investigated in the present study, while some other properties still remain to be evaluated, representing the limitations of the study. We are aware of the importance of them for a complete antimicrobial characterization of the AMP. Considering crucial role of biofilm mass in developing antibiotic resistance through acting as a barrier to prevent access of antibiotics to biofilm forming pathogens, there appears to be a need for designing therapeutic agents possessing ability to reduce or remove pre-existing biofilm. Accordingly, the lack of data on the efficacy of AMPs in removing pre-existing biofilm can be considered as a limitation of the current study.

In the present study in silico comparative sequence analysis and antimicrobial characterization led to describe a new, previously unrecognized antimicrobial function for named dicentracin-like peptide in comparison to moronecidin. This research really provides a descriptive report on functional characterization of the peptide. However potential mechanisms of antimicrobial action such as membrane leakiness, efflux of cell metabolites and inhibition of DNA synthesis need to be determined which can inform the design and conduct of future studies.

## Supporting information

S1 Fig Motifs and sequence of dicentracin-like precursor. (A): This peptide includes two motif pleurocidin and testis expressed sequence family that was defined by motif finder (http://www.genome.jp/tools/motif/). (B): The deduced cDNA and amino acid of dicentracin-like precursor, red residue represent mature dicentracin-like.(C): Figure represent phylogenic tree for mature Dicentracin-like and others mature piscines. HKPLP, pleurocidin-like peptide from *Hippocampus kudaBleeker*; TP-3, tilapia Piscidin 3; CATH-5, cathelicidins from *Sarcophilusharrisii*.
(TIF)

S2 Fig The alignment of amino acid sequence of dicentracin-like precursor with other piscine precursor. Dicentracin-like (XP_018543833.1, *Latescalcarifer*); Piscidin-1 or moronecidin (Q8UUG0.1, *Morone saxatilis*); PiscidinPiscidin-2 (ADY86111.1, *Epinepheluscoioides*(; PiscidinPiscidin-3 (AKA60776.1, *Epinepheluscoioides*); PiscidinPiscidin-4 (AKA60777.2, *Epinepheluscoioides*); PiscidinPiscidin-5 (APQ32052.1, *Moronechrysops*); PiscidinPiscidin 6 (APQ32044.1, *Morone chrysops*); PiscidinPiscidin 7 (APQ32054.1, *Moronesaxatilis*); Piscidin (ARK85994.1, *Seriola lalandi*(; Pleurocidin (P81941.2, *Pseudopleuronectesamericanus*); Piscidin-1 precursor (ACS91329.1, *Gadusmorhua*); Piscidin-2 precursor (ADU34222.1 *Gadusmorhua*); Piscidin-like protein (AGN52988.1, *Larimichthyscrocea*).
(TIF)

S3 Fig Expression analysis of dicentracin-like gene in various tissues. The expression of dicentracin-like gene was assayed in tissue including skin, head kidney, gill and intestineby reverse transcriptase-PCR (RT-PCR).D, dicentracin-like, EF1α gene, elongation factor 1 alpha.
(TIF)
