# Correction: Identification of novel antimicrobial peptide from Asian sea bass (*Lates calcarifer*) by *in silico* and activity characterization

**DOI:** 10.1371/journal.pone.0212759

**Published:** 2019-02-20

**Authors:** Behrouz Taheri, Mohsen Mohammadi, Iraj Nabipour, Niloofar Momenzadeh, Mona Roozbehani

The following information is missing from the Funding section: This study was also supported by the Iran National Science Foundation Research Chair Award No. 95/INSF/44913.
