# Supplementary figures and images for: Identification of novel antimicrobial peptide from Asian sea bass (Lates calcarifer) by in silico and activity characterization
Source: PLoS One. 2018 Oct 26;13(10):e0206578. doi: 10.1371/journal.pone.0206578 (PMC6203393; doi:10.1371/journal.pone.0206578)

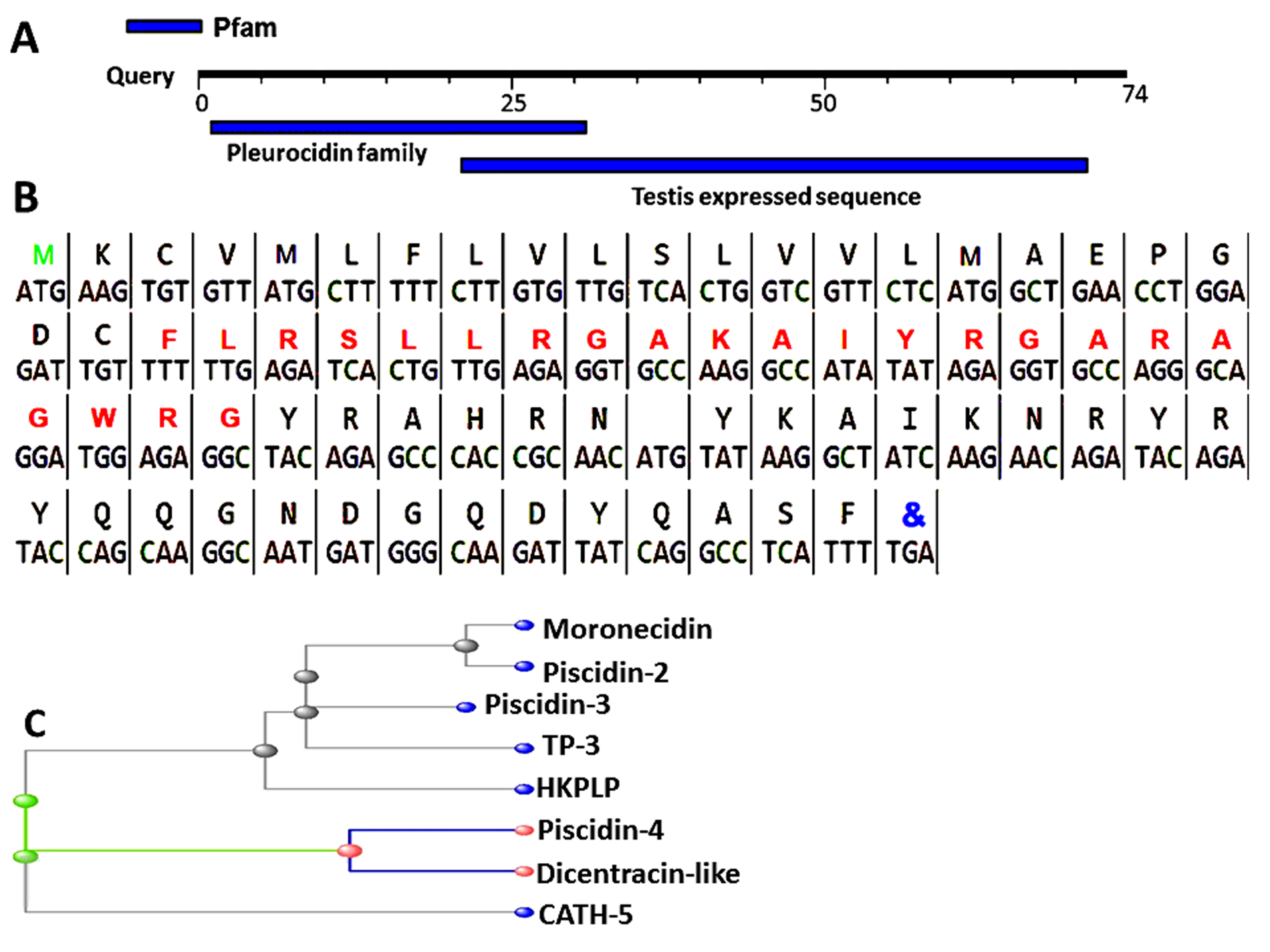

Supplement: S1 Fig — (A): This peptide includes two motif pleurocidin and testis expressed sequence family that was defined by motif finder (http://www.genome.jp/tools/motif/). (B): The deduced cDNA and amino acid of dicentracin-like precursor, red residue represent mature dicentracin-like.(C): Figure represent phylogenic tree for mature Dicentracin-like and others mature piscines. HKPLP, pleurocidin-like peptide from Hippocampus kudaBleeker; TP-3, tilapia Piscidin 3; CATH-5, cathelicidins from Sarcophilusharrisii. (TIF) [file pone.0206578.s001.tif]

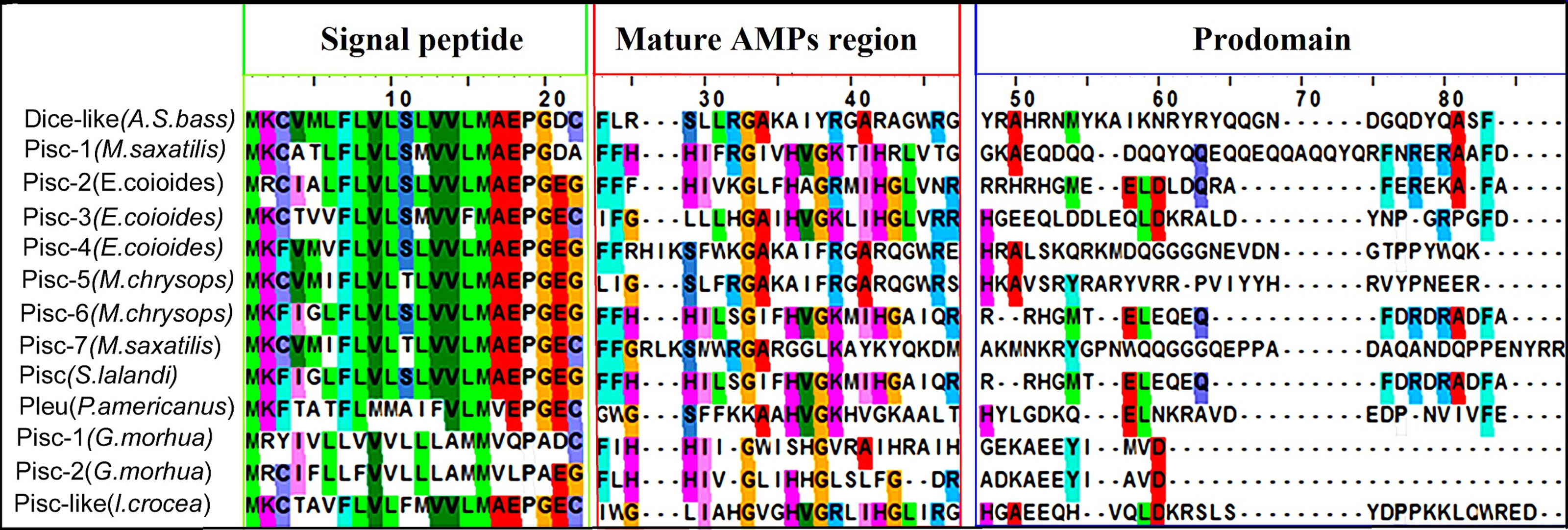

Supplement: S2 Fig — Dicentracin-like (XP_018543833.1, Latescalcarifer); Piscidin-1 or moronecidin (Q8UUG0.1, Morone saxatilis); PiscidinPiscidin-2 (ADY86111.1, Epinepheluscoioides(; PiscidinPiscidin-3 (AKA60776.1, Epinepheluscoioides); PiscidinPiscidin-4 (AKA60777.2, Epinepheluscoioides); PiscidinPiscidin-5 (APQ32052.1, Moronechrysops); PiscidinPiscidin 6 (APQ32044.1, Morone chrysops); PiscidinPiscidin 7 (APQ32054.1, Moronesaxatilis); Piscidin (ARK85994.1, Seriola lalandi(; Pleurocidin (P81941.2, Pseudopleuronectesamericanus); Piscidin-1 precursor (ACS91329.1, Gadusmorhua); Piscidin-2 precursor (ADU34222.1 Gadusmorhua); Piscidin-like protein (AGN52988.1, Larimichthyscrocea). (TIF) [file pone.0206578.s002.tif]

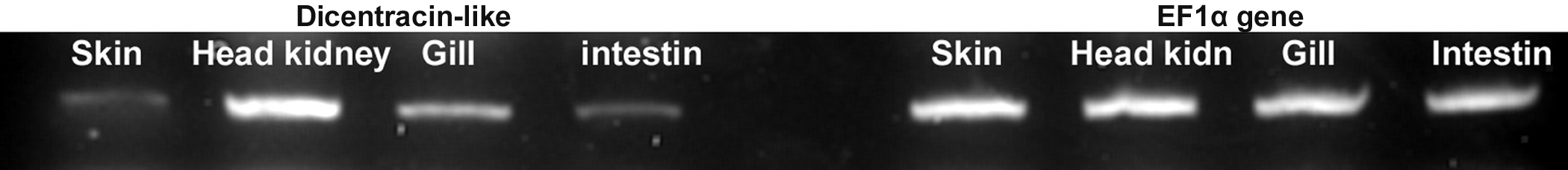

Supplement: S3 Fig — The expression of dicentracin-like gene was assayed in tissue including skin, head kidney, gill and intestineby reverse transcriptase-PCR (RT-PCR).D, dicentracin-like, EF1α gene, elongation factor 1 alpha. (TIF) [file pone.0206578.s003.tif]
